# Identification of an X-Band Clock Transition in Cp′_3_Pr^–^ Enabled by a 4f^2^5d^1^ Configuration

**DOI:** 10.1021/jacs.3c12725

**Published:** 2024-02-22

**Authors:** Patrick W. Smith, Jakub Hrubý, William J. Evans, Stephen Hill, Stefan G. Minasian

**Affiliations:** † Lawrence Berkeley National Laboratory, One Cyclotron Road, Berkeley, California 94720, United States; ‡ National High Magnetic Field Laboratory, 1800 East Paul Dirac Drive, Tallahassee, Florida 32310, United States; § Department of Chemistry, University of California, Irvine, Irvine, California 92697, United States; ∥ Department of Physics, Florida State University, Tallahassee, Florida 32306, United States

## Abstract

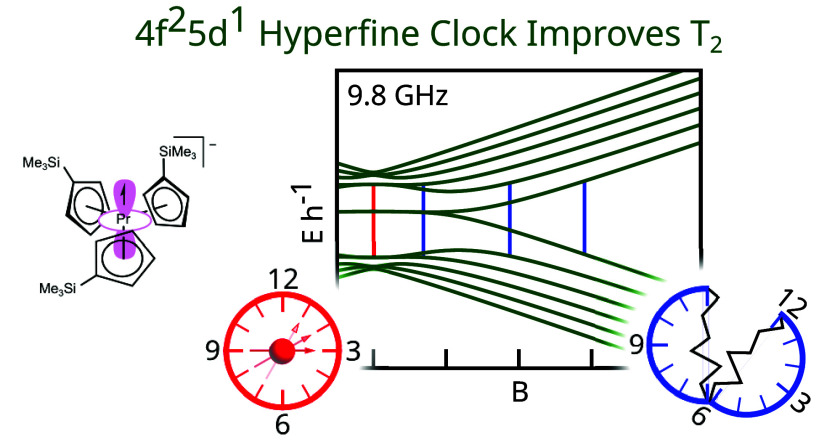

Molecular qubits offer an attractive basis for quantum information processing, but challenges remain with regard to sustained coherence. Qubits based on clock transitions offer a method to improve the coherence times. We propose a general strategy for identifying molecules with high-frequency clock transitions in systems where a d electron is coupled to a crystal-field singlet state of an f configuration, resulting in an *M*_*J*_ = ±1/2 ground state with strong hyperfine coupling. Using this approach, a 9.834 GHz clock transition was identified in a molecular Pr complex, [K(crypt)][Cp′_3_Pr^II^], leading to 3-fold enhancements in *T*_2_ relative to other transitions in the spectrum. This result indicates the promise of the design principles outlined here for the further development of f-element systems for quantum information applications.

The ability to manipulate coherence in a quantum object enables its use as a quantum bit (“qubit”). This is key to the development of new concepts in quantum information science (QIS), including sensing, communication, and computing.^1−9^ High-performance systems based on trapped ions^10−15^ and superconducting qubits^16−19^ have long coherence times but offer limited scalability and tunability. Recent advances have shown that molecular qubit designs may provide the necessary means for control and functionality, but they are also inherently more coupled to the environment, which tends to diminish performance. Variations of the ligand can provide opportunities to address this shortcoming; however, the principles needed to rationally design new molecular qubits with targeted properties have not been fully developed. Coherence times in molecular qubits can be improved by magnetic dilution^20,21^ and elimination of atoms with nuclear moments within the molecule.^22,23^ Limitations to both of these approaches make it desirable to explore alternatives.

Coherence times in electron-spin molecular qubits are improved at avoided energy-level crossings where the dependence of the transition frequency (ν) on the magnetic field (*B*) vanishes (i.e., dν/d*B* = 0). As such, the transverse relaxation time (*T*_2_) is less sensitive to magnetic noise, engendering resistance to decoherence from nearby magnetic sites^24^ and other nuclear spins in the molecule.^25^ Avoided crossings can be generated by nondiagonal perturbations of the spin Hamiltonian such as crystal-field-splitting^24^ and hyperfine^25−27^ interactions. For lanthanide systems, hyperfine clock qubits have only been realized with La^II^ (4f^0^5d^1^) and Lu^II^ (4f^14^5d^1^)^25^ centers. These systems have closed f shells, which minimize anisotropy, but this is possible only at the beginning and end of the lanthanide series. There is a significant question of whether coherence enhancements can be realized at hyperfine-derived clock transitions in open-f-shell systems.

Here we provide a framework for identifying clock qubit candidates based on open-shell 4f^*n*^5d^1^ configurations of f elements with even *n*. Our approach was to identify trivalent 4f^*n*^ complexes with crystal fields (CFs) that enforce singlet (*M*_*J*_ = 0) ground states (GSs). We hypothesized that reduction to the divalent 4f^*n*^5d^1^ complex would afford an *M*_*J*_ = ±1/2 GS doublet in the M^II^ complex. Open-shell-singlet GSs are well-known in actinide chemistry, with precedent in 5f^2^, 5f^4^, and 5f^8^ systems.^28^ Equatorial ligand fields provide an additional advantage by promoting occupation of a 5d–6s hybrid orbital,^29−38^ where d–s hybridization in this singly-occupied molecular orbital increases Fermi contact and induces massive hyperfine couplings,^39^ bringing clock transitions to higher frequency. Both of these criteria were met by Cp′_3_Pr^III^ (**1**) (Cp′ = trimethylsilylcyclopentadienyl), which has a singlet GS, as evidenced in published magnetometry data^29^ by the limiting value near 0 for χ*T* at low temperatures. These data also indicate a 4f^2^5d^1^ configuration for divalent [K(crypt)][Cp′_3_Pr^II^] (**2**) (crypt = [2.2.2]cryptand). Combined with the large hyperfine coupling^40,41^ and 100% natural abundance of ^141^Pr, the Cp′_3_Pr system is ideal for testing the hypothesis described above.
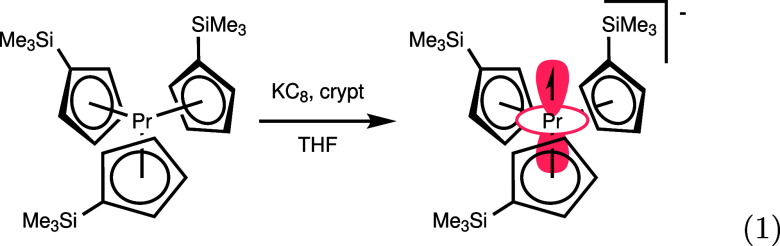
1

While the published magnetometry data for **1** were consistent with a singlet GS, in the *C*_3*h*_ pseudosymmetry of the molecule there are in fact three possible singlet states with *M*_*J*_ = 0 and (|+3⟩ ± |−3⟩)/√2, where the latter pair is generated by the *B*_6_^6^ component of the CF. Fortuitously, CF modeling of optical data has been reported for **1**,^42^ indicating that the ground state is *M*_*J*_ = 0. We have further confirmed this by fitting^43^ the reported magnetometry data to the model Hamiltonian in eq 2:
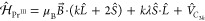
2with Zeeman, spin–orbit, and CF terms (see section S2.1 in the Supporting Information for a full discussion). The slight differences in CF parameters relative to those previously reported (Table 1) are most likely due to the more simplistic model used for the present treatment. These parameters afforded the state diagram shown in Figure 1, indicating that the GS of this molecule is |*J*, *M*_*J*_⟩ = |4, 0⟩, which is separated from |4, ±1⟩ by 190 cm^–1^.

**Table 1:** CF Parameters for **1** and **2** Determined by Fitting Magnetometry Data Reported in Reference (29); The Optical CF Parameters in the First Column Are Taken from Reference (42)^a^

	**1** (opt.)	**1** (mag.)	**2**^b^
*B*_2_^0^	–2485	–3129(19)	–810(60)
*B*_4_^0^	1323	1980(20)	1230(40)
*B*_6_^0^	555	484(11)	484^c^
*B*_6_^6^	–1956	–1760(60)	–1760^c^
*k*	–	0.98^c^	0.98^c^

a All CF values are in units of cm^–1^. λ was fixed at 380 cm^–1^, which is 90% of the optimized free ion value.^43^

b Fit includes an empirical TIP component of 7.4 × 10^–4^ emu mol^–1^. The exchange coupling, *j*, was set to 10,000 cm^–1^.

c Fixed during the fit.

**Figure 1:**
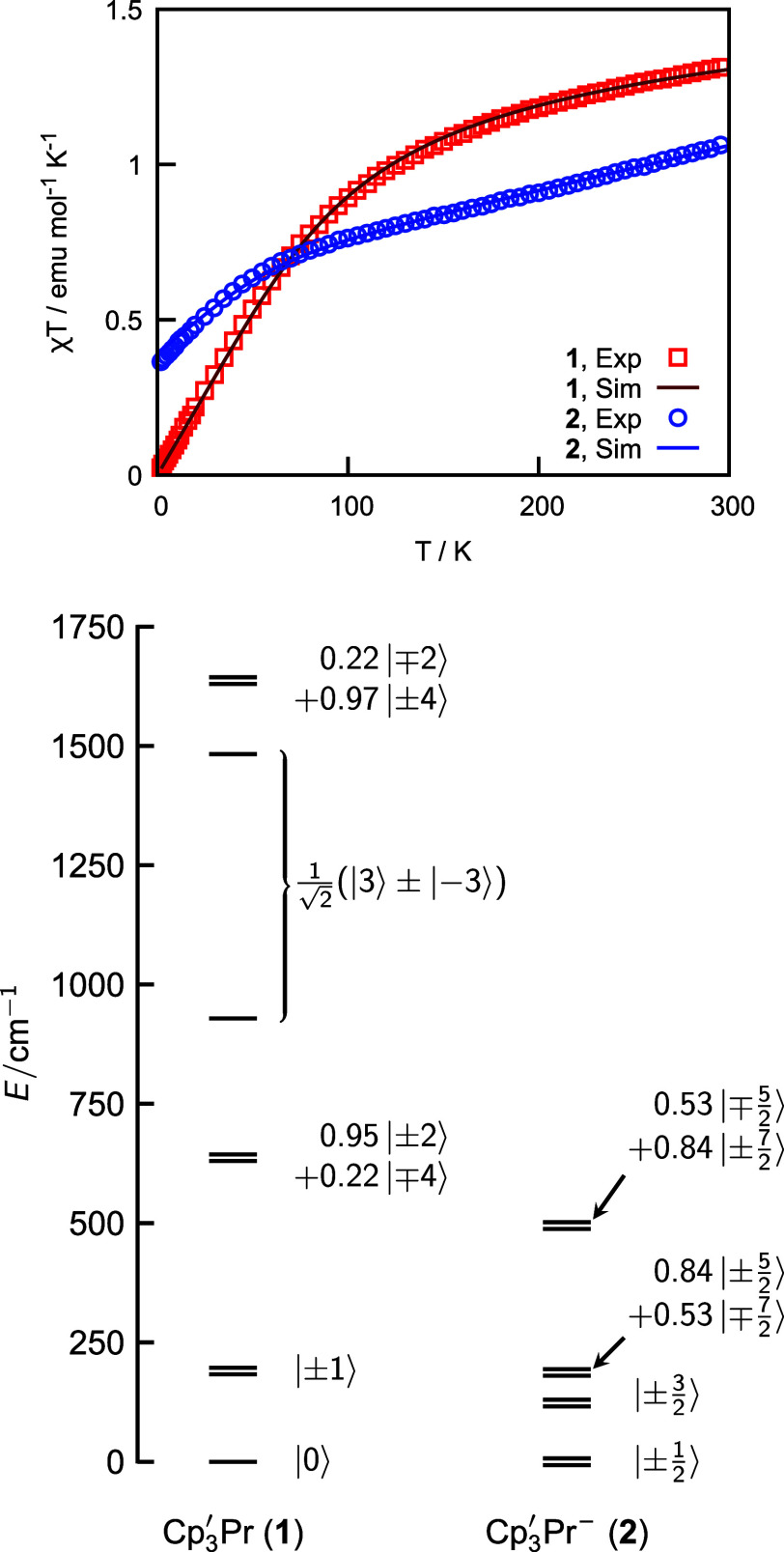
(top) Best fits of eqs 2 and 3 to the published magnetometry data^29^ for **1** and **2**, respectively. (bottom) Empirical state-energy diagrams for the ground multiplets of **1** (^3^H_4_, left) and **2** (^4^H_7/2_, right).

We next set out to model the magnetometry for **2** to assess the effect of the d electron on the state ordering. The reduced high-temperature magnetic moment of **2** relative to **1** is consistent with an LS coupling scheme,^29,44^ and eq 2 may be used to model the magnetometry data for **2** with *S* = 3/2 and *L* = 5, affording an *M*_*J*_ = ±1/2 GS of ^4^H_7/2_ parentage (see section S2.2 for fit details). However, the CF treatment in this model is unrealistic, as *V̂*_CF_ operates on all three electrons.

Instead, we elected to model the data by coupling the 4f angular momentum to a second *S* = 1/2 spin system representing the 5d–6s hybrid electron. This Hamiltonian is shown in eq 3:
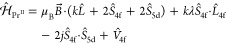
3where *V̂*_4f_ indicates the CF term acting only on the 4f electrons and *j* is a large, positive, isotropic exchange coupling describing the interaction between the 4f and 5d electron spins. This model has the advantage of allowing a direct comparison of 4f CF parameters between **1** and **2**. We assumed that the 4f CF was only perturbed by the additional 5d_*z*^2^_ electron, which should impact only the *B*_2_^0^ and *B*_4_^0^ parameters of the 4f CF Hamiltonian (section S2.3). These parameters and a residual temperature-independent paramagnetism (TIP) were the only parameters allowed to vary from those of **1** (section S2.2). The results of this fit to the magnetometry data for **2** are shown in Figure 1, and the best-fit parameters are shown in Table 1. Crucially, the GS from both models can be assigned as |7/2, ±1/2⟩, consistent with a conserved 4f state ordering relative to **1**. The reduction of the *B*_2_^0^ and *B*_4_^0^ CF parameters and smaller separation between the ground and first excited state (120 cm^–1^ vs 190 cm^–1^) indicate that the CF potential felt by the 4f electron is weakened upon addition of the 5d electron. This result is predictable, as the 5d electron itself responds to the ligand field and thus occupies orbitals that generate an antipodal potential with respect to that of the ligands.

Following confirmation of the *M*_*J*_ = ±1/2 GS in **2**, we set out to characterize its hyperfine and relaxation behavior using X-band EPR spectroscopy on a magnetically dilute powder of ca. 1% **2** in [K(crypt)][Cp′_3_Yb^II^] (**2**_**Yb**_), which is diamagnetic due to a 4f^14^ configuration.^30,31^ While no spectral features attributable to **2** were observed using continuous-wave (cw) detection, it was possible to collect an echo-detected field-swept (EDFS) spectrum of **2** (Figure 2a). The spectrum was integrated across a range of delay times to suppress distortion due to electron spin echo envelope modulation (ESEEM) from ^1^H hyperfine coupling.^45^ This X-band spectrum is highly complex and cannot be immediately attributed to a spin system with pseudoaxial symmetry and hyperfine coupling to a ^141^Pr nucleus. However, this spectrum could be consistent with **2** in the weak-field limit,^39^ a possibility that we probed using higher-frequency EPR spectroscopy.

**Figure 2:**
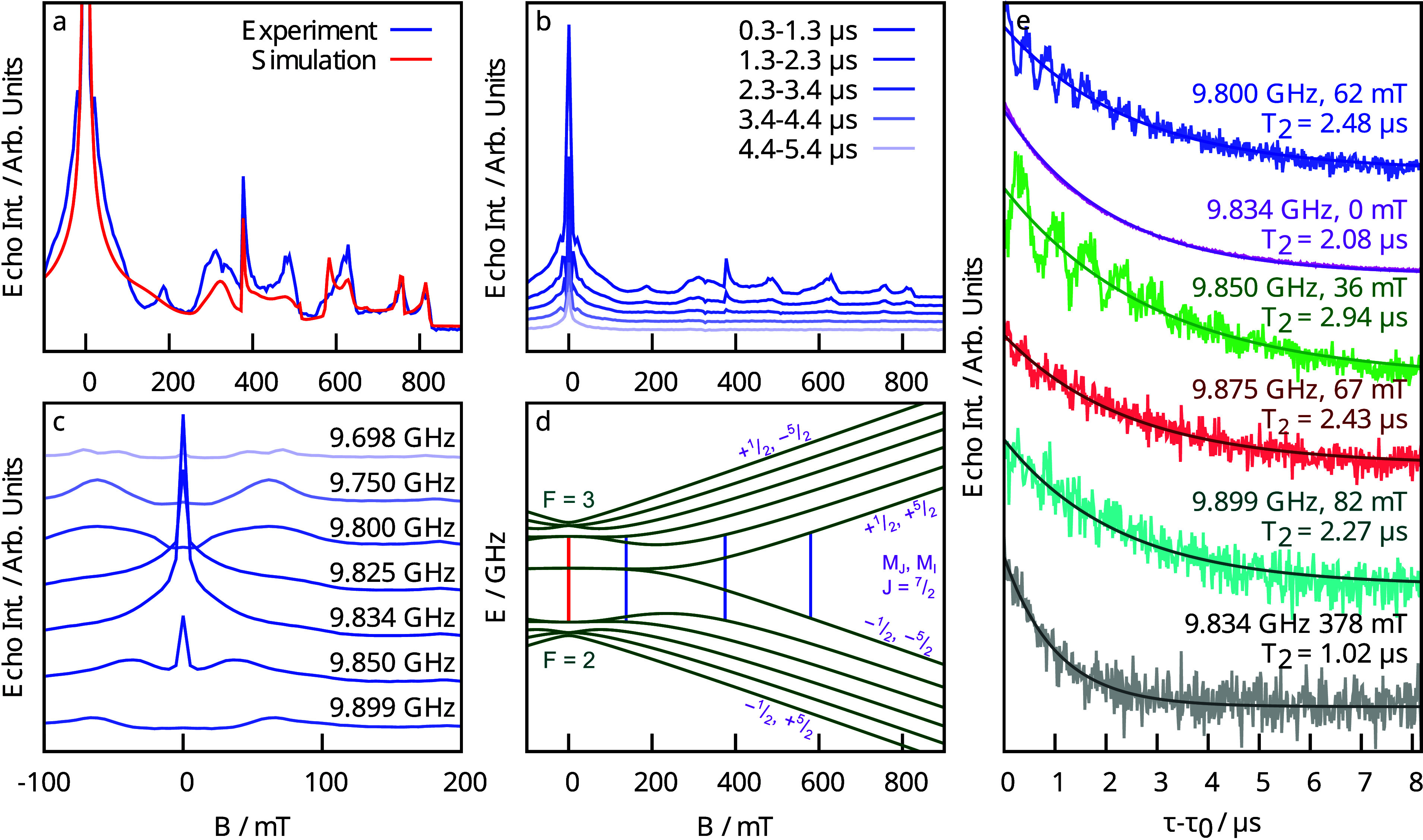
X-band EPR spectroscopy of 1% **2** in **2**_**Yb**_ at 5 K. (a) EDFS X-band EPR spectrum of **2** (integrated over ca. 1 μs delay times) together with a simulated spectrum based on the parameters in Table 2. (b) Variable-delay EDFS spectra of **2** showing the more rapid decay of spectral components above about 100 mT. (c) Variable-frequency EDFS spectra integrated over 8 μs delays showing a sharp transition at zero field and 9.834 GHz; spectra were mirrored around zero field for clarity. (d) Empirical state energy diagram for the ⊥ orientation of **2** showing allowed transitions at 9.834 GHz in blue. The clock transition is highlighted in red. (e) Primary echo decay curves at various frequencies and fields from 0 to 100 mT together with results of fitting exponential decays to determine *T*_2_. A decay at 378 mT is shown for comparison.

The 70 GHz spectrum of an undiluted powder of **2** is shown in Figure 3 together with a simulated spectrum. In contrast to the X-band results, this spectrum is pseudoaxial with a clear hyperfine coupling, enabling the modeling of the *g⃡* and *A⃡* tensors. Analysis of both X-band and the high-frequency EPR spectra of **2** is based on the effective spin Hamiltonian (SH)^46^ in eq 4 using a pseudospin (*S* = 1/2) system coupled to an *I* = 5/2 nuclear spin:
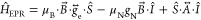
4with electron and nuclear Zeeman contributions and hyperfine terms (see section S5.1).

**Figure 3:**
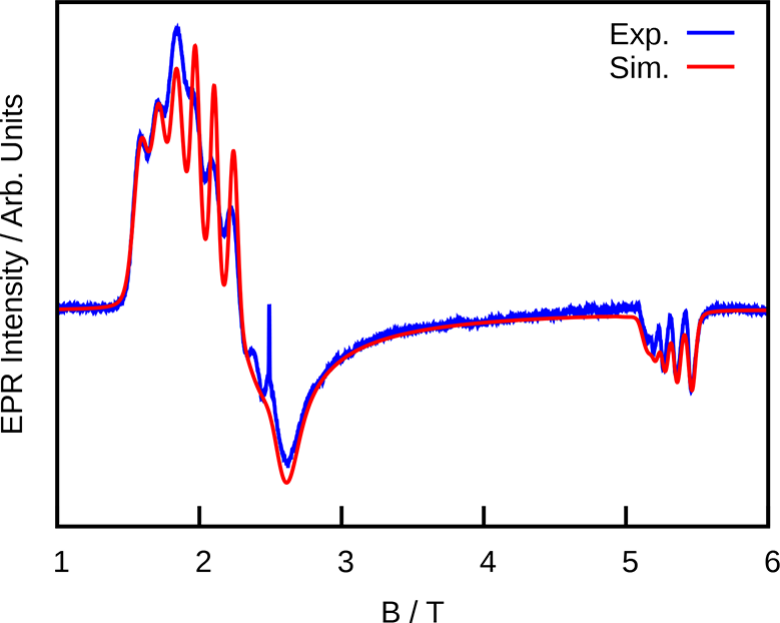
Simulation (red) and experimental 70 GHz cw EPR spectrum (blue) of an undiluted powder of **2** at 5 K. The small sharp peak at ca. 2.5 T is a minor *g* = 2 impurity.

The SH parameters from the 70 GHz spectrum (Table 2) enabled modeling of the X-band spectrum using eq 4. In this case, the spectrum could be modeled using an axial SH, with the *g*_⊥_ and *A*_⊥_ values near the average of *g*_*x*,*y*_ and *A*_*x*,*y*_ extracted from the 70 GHz data. The higher apparent symmetry at X-band may be due to the presence of near-neighbor magnetic sites in the concentrated sample, which may be sufficient to desymmetrize the SH. These *g* values are somewhat different from those predicted for a ^4^H_7/2_*M*_*J*_ = ±1/2 GS (*g*_⊥_ = 2.67, *g*_∥_ = 0.67) but are in good agreement with those predicted by PHI^43^ using the model based on eq 3 (*g*_⊥_ = 2.43, *g*_∥_ = 0.81), supporting our assignment of the GS.

**Table 2:** Parameters from Simulating Multifrequency EPR to a Rhombic (70 GHz) or Axial (9.834 GHz) Pseudospin Hamiltonian and *g* Values Predicted by the Two Spin Hamiltonians for an *M*_*J*_ = ±1/2 Doublet

ν or		⊥		∥
70^a^	*g*	2.60	2.21	0.94
	*A*	4.70^a^	3.70^a^	0.87^a^
9.834^a^	*g*	2.43		0.94
	*A*	4.33^a^		0.87^a^
(^4^H_7/2_)	*g*	2.67		0.67
	*g*	2.42		0.81

a In GHz.

The state energy versus magnetic field diagram from this model (Figure 2d) has an avoided energy level crossing generated by a nondiagonal term in the hyperfine coupling with a transition frequency of 9.834 GHz in the perpendicular orientation at low magnetic fields. Variable-frequency EPR spectroscopy shows a dramatic enhancement in signal intensity at 0 mT and 9.834 GHz (Figure 2c), and the primary echo decay shows no evidence of ESEEM. Both of these observations are consistent with a clock transition,^47^ although the vanishing ^1^H Larmor frequency at zero field may also contribute to the lack of ESEEM. Intriguingly, all of the features in the range of 0–100 mT have decay constants in the range of 2–3 μs, while the higher-field transitions, which arise from Zeeman splitting, have *T*_2_ times closer to 1 μs (Figure 2b,e). The longest relaxation times do not occur precisely at the zero-field clock transition but rather were observed slightly away at 9.850 GHz and 36 mT (*T*_2_ = 2.94 μs).

The origin of the *T*_2_ enhancement over this wide field range can be explained by the nearly field-independent nature of the state energy of the two |*F*, *M*_*F*_⟩ states involved in the relevant transitions (*F* = *J* + *I* is the combined electronic and nuclear angular momentum). In this range, a number of the parent |*M*_*J*_, *M*_*I*_⟩ states sequentially approach one another in energy, and mixing between them to form the final |*F*, *M*_*F*_⟩ states at low field leads to the extended weak dependence of the state energy on the field. These transitions invariably relax slower than those at higher fields (by a factor of 2–3), consistent with a clocklike enhancement of the relaxation due to the weak dependence of the frequency on the field. We additionally note that the *T*_1_ (longitudinal) relaxation times determined for **2** were also ca. 3 μs at 5 K (Figure S4), which may limit the degree to which *T*_2_ can be enhanced at the clock transition.

The combined magnetometry and EPR results presented above support the fundamental hypothesis that coupling of an open-shell-singlet 4f configuration to an electron in an s–d hybrid orbital results in an *M*_*J*_ = ±1/2 doublet, with large hyperfine couplings that give rise to high-frequency clock transitions with longer *T*_2_ relaxations. This was accomplished by enforcing a low-anisotropy 4f configuration, enabling performance that is normally possible only with the closed-4f-shell configurations at either end of the lanthanide series. Future work will focus on using these design principles to target new molecular hyperfine qubits based on lanthanide and actinide centers with both f and d electrons.

## Supporting Information Available

Experimental details, discussion of model development for eq 3, additional EPR spectroscopic data, and complete EPR simulation parameters (PDF)

## References

[ref1] Chuang I. L.; Yamamoto Y. Simple quantum computer. Phys. Rev. A 1995, 52, 3489 10.1103/PhysRevA.52.3489. 9912648

[ref2] Schliemann J.; Khaetskii A. V.; Loss D. Spin decay and quantum parallelism. Phys. Rev. B 2002, 66, 245303 10.1103/PhysRevB.66.245303.

[ref3] Arute F.; et al. Quantum supremacy using a programmable superconducting processor. Nature 2019, 574, 505–510. 10.1038/s41586-019-1666-5. 31645734

[ref4] Huang H. Y.; Broughton M.; Cotler J.; Chen S.; Li J.; Mohseni M.; Neven H.; Babbush R.; Kueng R.; Preskill J.; McClean J. R. Quantum advantage in learning from experiments. Science 2022, 376, 1182–1186. 10.1126/science.abn7293. 35679419

[ref5] Bennett C. H.; Brassard G.; Ekert A. K. Quantum cryptography. Sci. Am. 1992, 267, 50–57. 10.1038/scientificamerican1092-50.

[ref6] Gisin N.; Ribordy G.; Tittel W.; Zbinden H. Quantum cryptography. Rev. Mod. Phys. 2002, 74, 145 10.1103/RevModPhys.74.145.

[ref7] Perdomo-Ortiz A.; Dickson N.; Drew-Brook M.; Rose G.; Aspuru-Guzik A. Finding low-energy conformations of lattice protein models by quantum annealing. Sci. Rep. 2012, 2, 571 10.1038/srep00571. 22891157 PMC3417777

[ref8] Gershenfeld N.; Chuang I. L. Quantum Computing with Molecules. Sci. Am. 1998, 278, 66–71. 10.1038/scientificamerican0698-66.9418300

[ref9] Grover L. K. A fast quantum mechanical algorithm for database search. In Proceedings of the 28th Annual ACM Symposium on Theory of Computing, Philadelphia, PA, May 22–24, 1996; ACM, 1996; pp 212–219.

[ref10] Cirac J. I.; Zoller P. Quantum Computations with Cold Trapped Ions. Phys. Rev. Lett. 1995, 74, 4091–4094. 10.1103/PhysRevLett.74.4091. 10058410

[ref11] Monroe C.; Kim J. Scaling the ion trap quantum processor. Science 2013, 339, 1164–1169. 10.1126/science.1231298. 23471398

[ref12] Wang P.; Luan C. Y.; Qiao M.; Um M.; Zhang J.; Wang Y.; Yuan X.; Gu M.; Zhang J.; Kim K. Single ion qubit with estimated coherence time exceeding one hour. Nat. Commun. 2021, 12, 233 10.1038/s41467-020-20330-w. 33431845 PMC7801401

[ref13] Srinivas R.; Löschnauer C. M.; Malinowski M.; Hughes A. C.; Nourshargh R.; Negnevitsky V.; Allcock D. T. C.; King S. A.; Matthiesen C.; Harty T. P.; Ballance C. J. Coherent Control of Trapped Ion Qubits with Localized Electric Fields. Phys. Rev. Lett. 2023, 131, 020601 10.1103/PhysRevLett.131.020601. 37505962

[ref14] Maksymov A.; Nguyen J.; Chaplin V.; Nam Y.; Markov I. L. Detecting Qubit-Coupling Faults in Ion-Trap Quantum Computers. In 2022 IEEE International Symposium on High-Performance Computer Architecture (HPCA), Seoul, Korea, April 2–6, 2022; IEEE, 2022; pp 387–399.

[ref15] Akhtar M.; Bonus F.; Lebrun-Gallagher F. R.; Johnson N. I.; Siegele-Brown M.; Hong S.; Hile S. J.; Kulmiya S. A.; Weidt S.; Hensinger W. K. A high-fidelity quantum matter-link between ion-trap microchip modules. Nat. Commun. 2023, 14, 531 10.1038/s41467-022-35285-3. 36754957 PMC9908934

[ref16] Reed M. D.; DiCarlo L.; Nigg S. E.; Sun L.; Frunzio L.; Girvin S. M.; Schoelkopf R. J. Realization of three-qubit quantum error correction with superconducting circuits. Nature 2012, 482, 382–385. 10.1038/nature10786. 22297844

[ref17] Devoret M. H.; Schoelkopf R. J. Superconducting circuits for quantum information: An outlook. Science 2013, 339, 1169–1174. 10.1126/science.1231930. 23471399

[ref18] Zhang J.; Pagano G.; Hess P. W.; Kyprianidis A.; Becker P.; Kaplan H.; Gorshkov A. V.; Gong Z.-X.; Monroe C. Observation of a many-body dynamical phase transition with a 53-qubit quantum simulator. Nature 2017, 551, 601–604. 10.1038/nature24654. 29189781 PMC6506159

[ref19] Kim Y.; Eddins A.; Anand S.; Wei K. X.; van den Berg E.; Rosenblatt S.; Nayfeh H.; Wu Y.; Zaletel M.; Temme K.; Kandala A. Evidence for the utility of quantum computing before fault tolerance. Nature 2023, 618, 500–505. 10.1038/s41586-023-06096-3. 37316724 PMC10266970

[ref20] Yu C.-J.; von Kugelgen S.; Krzyaniak M. D.; Ji W.; Dichtel W. R.; Wasielewski M. R.; Freedman D. E. Spin and Phonon Design in Modular Arrays of Molecular Qubits. Chem. Mater. 2020, 32, 10200–10206. 10.1021/acs.chemmater.0c03718.

[ref21] Bader K.; Dengler D.; Lenz S.; Endeward B.; Jiang S.-D.; Neugebauer P.; van Slageren J. Room temperature quantum coherence in a potential molecular qubit. Nat. Commun. 2014, 5, 5304 10.1038/ncomms6304. 25328006

[ref22] Graham M. J.; Yu C.-J.; Krzyaniak M. D.; Wasielewski M. R.; Freedman D. E. Synthetic Approach To Determine the Effect of Nuclear Spin Distance on Electronic Spin Decoherence. J. Am. Chem. Soc. 2017, 139, 3196–3201. 10.1021/jacs.6b13030. 28145700

[ref23] Zadrozny J. M.; Niklas J.; Poluektov O. G.; Freedman D. E. Millisecond coherence time in a tunable molecular electronic spin qubit. ACS Cent. Sci. 2015, 1, 488–492. 10.1021/acscentsci.5b00338. 27163013 PMC4827467

[ref24] Shiddiq M.; Komijani D.; Duan Y.; Gaita-Ariño A.; Coronado E.; Hill S. Enhancing coherence in molecular spin qubits via atomic clock transitions. Nature 2016, 531, 348–351. 10.1038/nature16984. 26983539

[ref25] Kundu K.; White J. R.; Moehring S. A.; Yu J. M.; Ziller J. W.; Furche F.; Evans W. J.; Hill S. A 9.2-GHz clock transition in a Lu(II) molecular spin qubit arising from a 3,467-MHz hyperfine interaction. Nat. Chem. 2022, 14, 392–397. 10.1038/s41557-022-00894-4. 35288686

[ref26] Wolfowicz G.; Tyryshkin A. M.; George R. E.; Riemann H.; Abrosimov N. V.; Becker P.; Pohl H.-J.; Thewalt M. L. W.; Lyon S. A.; Morton J. J. L. Atomic clock transitions in silicon-based spin qubits. Nat. Nanotechnol. 2013, 8, 561–564. 10.1038/nnano.2013.117. 23793304

[ref27] Zadrozny J. M.; Gallagher A. T.; Harris T. D.; Freedman D. E. A Porous Array of Clock Qubits. J. Am. Chem. Soc. 2017, 139, 7089–7094. 10.1021/jacs.7b03123. 28453274

[ref28] Edelstein N. M.; Lander G. H. In The Chemistry of the Actinide and Transactinide Elements; Morss L. R., Edelstein N. M., Fuger J., Eds.; Springer: Dordrecht, The Netherlands, 2011; pp 2225–2306.

[ref29] Meihaus K. R.; Fieser M. E.; Corbey J. F.; Evans W. J.; Long J. R. Record High Single-Ion Magnetic Moments Through 4f^n^5d^1^ Electron Configurations in the Divalent Lanthanide Complexes [(C_5_H_4_SiMe_3_)_3_Ln]^−^. J. Am. Chem. Soc. 2015, 137, 9855–9860. 10.1021/jacs.5b03710. 26168303

[ref30] MacDonald M. R.; Bates J. E.; Ziller J. W.; Furche F.; Evans W. J. Completing the Series of + 2 Ions for the Lanthanide Elements: Synthesis of Molecular Complexes of Pr^2+^, Gd^2+^, Tb^2+^, and Lu^2+^. J. Am. Chem. Soc. 2013, 135, 9857–9868. 10.1021/ja403753j. 23697603

[ref31] Fieser M. E.; MacDonald M. R.; Krull B. T.; Bates J. E.; Ziller J. W.; Furche F.; Evans W. J. Structural, spectroscopic, and theoretical comparison of traditional vs recently discovered Ln^2+^ ions in the [K(2.2.2-cryptand)][(C_5_H_4_SiMe_3_)_3_Ln] complexes: The variable nature of Dy^2+^ and Nd^2+^. J. Am. Chem. Soc. 2015, 137, 369–382. 10.1021/ja510831n. 25541886

[ref32] Windorff C. J.; MacDonald M. R.; Meihaus K. R.; Ziller J. W.; Long J. R.; Evans W. J. Expanding the Chemistry of Molecular U^2+^ Complexes: Synthesis, Characterization, and Reactivity of the {[C_5_H_3_(SiMe_3_)_2_]_3_U}^−^ Anion. Chem. - Eur. J. 2016, 22, 772–782. 10.1002/chem.201503583. 26636775

[ref33] Fieser M. E.; et al. Evaluating the electronic structure of formal Ln^II^ ions in Ln^II^(C_5_H_4_SiMe_3_)_3_^1–^ using XANES spectroscopy and DFT calculations. Chem. Sci. 2017, 8, 6076–6091. 10.1039/C7SC00825B. 28989638 PMC5625586

[ref34] Fieser M. E.; Palumbo C. T.; La Pierre H. S.; Halter D. P.; Voora V. K.; Ziller J. W.; Furche F.; Meyer K.; Evans W. J. Comparisons of lanthanide/actinide +2 ions in a tris(aryloxide)arene coordination environment. Chem. Sci. 2017, 8, 7424–7433. 10.1039/C7SC02337E. 29163894 PMC5674182

[ref35] Su J.; Windorff C. J.; Batista E. R.; Evans W. J.; Gaunt A. J.; Janicke M. T.; Kozimor S. A.; Scott B. L.; Woen D. H.; Yang P. Identification of the Formal +2 Oxidation State of Neptunium: Synthesis and Structural Characterization of {Np^II^[C_5_H_3_(SiMe_3_)_2_]_3_}^1–^. J. Am. Chem. Soc. 2018, 140, 7425–7428. 10.1021/jacs.8b03907. 29870238

[ref36] Ryan A. J.; Darago L. E.; Balasubramani S. G.; Chen G. P.; Ziller J. W.; Furche F.; Long J. R.; Evans W. J. Synthesis, Structure, and Magnetism of Tris(amide) [Ln{N(SiMe_3_)_2_}_3_]^1–^ Complexes of the Non-traditional + 2 Lanthanide Ions. Chem. - Eur. J. 2018, 24, 7702–7709. 10.1002/chem.201800610. 29490123

[ref37] Fang M.; Lee D. S.; Ziller J. W.; Doedens R. J.; Bates J. E.; Furche F.; Evans W. J. Synthesis of the (N_2_)^3–^ Radical from Y^2+^ and Its Protonolysis Reactivity To Form (N_2_H_2_)^2–^ via the Y[N(SiMe_3_)_2_]_3_/KC_8_ Reduction System. J. Am. Chem. Soc. 2011, 133, 3784–3787. 10.1021/ja1116827. 21361288

[ref38] Woen D. H.; Chen G. P.; Ziller J. W.; Boyle T. J.; Furche F.; Evans W. J. Solution Synthesis, Structure, and CO_2_ Reduction Reactivity of a Scandium(II) Complex, {Sc[N(SiMe_3_)_2_]_3_}^−^. Angew. Chem. 2017, 129, 2082–2085. 10.1002/ange.201611758. 28097771

[ref39] Abragam A.; Bleaney B. Electron Paramagnetic Resonance of Transition Ions; Clarendon Press: Oxford, U.K., 1970.

[ref40] Bleaney B. Hyperfine Interactions in Rare-Earth Metals. J. Appl. Phys. 1963, 34, 1024–1031. 10.1063/1.1729355.

[ref41] Morton J.; Preston K. Atomic parameters for paramagnetic resonance data. J. Magn. Reson. (1969) 1978, 30, 577–582. 10.1016/0022-2364(78)90284-6.

[ref42] Jank S; Reddmann H; Amberger H.-D. The electronic structure of organometallic complexes of the f elements: XL. Crystal field strength of η^5^-cyclopentadienyl ligand estimated on the basis of the crystal field parameters of (Me_3_SiC_5_H_4_)_3_Pr^III^. J. Alloys Compd. 1997, 250, 387–390. 10.1016/S0925-8388(96)02554-6.

[ref43] Chilton N. F.; Anderson R. P.; Turner L. D.; Soncini A.; Murray K. S. PHI: A powerful new program for the analysis of anisotropic monomeric and exchange-coupled polynuclear d- and f-block complexes. J. Comput. Chem. 2013, 34, 1164–1175. 10.1002/jcc.23234. 23386394

[ref44] Anderson D. M.; Cloke F. G. N.; Cox P. A.; Edelstein N.; Green J. C.; Pang T.; Sameh A. A.; Shalimoff G. On the stability and bonding in bis(η-arene)ianthanide complexes. J. Chem. Soc., Chem. Commun. 1989, 53–55. 10.1039/C39890000053.

[ref45] Rowan L. G.; Hahn E. L.; Mims W. B. Electron-Spin-Echo Envelope Modulation. Phys. Rev. 1965, 137, A61 10.1103/PhysRev.137.A61.

[ref46] Stoll S.; Schweiger A. EasySpin, a comprehensive software package for spectral simulation and analysis in EPR. J. Magn. Reson. 2006, 178, 42–55. 10.1016/j.jmr.2005.08.013. 16188474

[ref47] Kundu K.; Chen J.; Hoffman S.; Marbey J.; Komijani D.; Duan Y.; Gaita-Ariño A.; Stanton J.; Zhang X.; Cheng H.-P.; Hill S. Electron-nuclear decoupling at a spin clock transition. Commun. Phys. 2023, 6, 38 10.1038/s42005-023-01152-w.

