# Supplementary material for: Identification of an X-Band Clock Transition in Cp′3Pr– Enabled by a 4f25d1 Configuration
Source: J Am Chem Soc. 2024 Feb 22;146(9):5781–5. doi: 10.1021/jacs.3c12725 (PMC10921394; doi:10.1021/jacs.3c12725)
Supplement: Supplementary file 1 — ja3c12725_si_001.pdf [file ja3c12725_si_001.pdf]

Supporting Information For: Identification of an X-Band Clock Transition in Cp'_3Pr^- Enabled by a $4\,f^2\,5\,d^1$ Configuration

Patrick W. Smith,[†] Jakub Hrubý,[‡] William J. Evans,[¶] Stephen Hill,[*,‡,§] and Stefan G. Minasian[*,†]

†*Lawrence Berkeley National Laboratory, One Cyclotron Rd., Berkeley, California 94720, United States*

‡*National High Magnetic Field Laboratory, 1800 E. Paul Dirac Drive Tallahassee, Florida 32310, United States*

¶*Department of Chemistry, University of California, Irvine, Irvine, California 92697, United States*

§*Department of Physics, Florida State University, Tallahassee, Florida 32306, United States*

E-mail: shill@magnet.fsu.edu; sgminasian@lbl.gov

Contents

1 Synthesis **3**

1.1 General Considerations . . . 3

1.2 Synthesis of [K(crypt)][Cp'_3Pr^{II}] (**2**) . . . 3

1.3 Preparation of Magnetically Dilute **2** . . . 3

2 Modeling of SQUID Magnetometry Data **4**

2.1 Crystal-Field Fitting of Magnetometry Data for **1** . . . 4

2.2 Crystal-Field Fitting of Magnetometry Data for **2** . . . 5

2.3 Crystal Field Approximation for $5d_{z^2}$ Acting on a 4f Shell . . . 7

3 X-band EPR Spectroscopy **10**

3.1 EDFS spectra and variable-field two-pulse ESEEM . . . 10

3.2 Relaxation time determinations . . . 11

4 HF-EPR Spectroscopy **11**

5 EPR Simulation **15**

5.1 Simulation of 70 GHz Continuous-Wave Spectra . . . 15

5.2 Simulation of X-band Spectra . . . 18

1 Synthesis

1.1 General Considerations

All handling was performed under Ar atmospheres using standard glovebox techniques. Tetrahydrofuran (THF) was dried over sodium benzophenone, distilled, and stored over 4 Å molecular sieves prior to use. Et_2O was degassed by sparging with Ar and purified by passage through a column of activated alumina, then stored over 4 Å molecular sieves prior to use. [K(crypt)][Cp'_3Yb^{II}] was prepared by reported procedures.[1]

1.2 Synthesis of [K(crypt)][Cp'_3Pr^{II}] (2)

Compound **2** was prepared by modification of an existing literature procedure[2] to enable a single-pot synthesis. $KC_5H_4SiMe_3$ (0.200 g, 1.130 mmol) was dissolved in THF (4 mL) and added to a suspension of $PrI_3(THF)_4$ (0.300 g, 0.376 mmol) in THF (2 mL). After stirring for 16 h, the resulting suspension was filtered onto 2.2.2-cryptand (0.143 g, 0.380 mmol) and cooled to −35 °C. KC_8 (0.052 g, 0.385 mmol) was added, the resulting suspension stirred for 5 min, then 4 mL Et_2O was added and the suspension was filtered. After layering with 10 mL Et_2O the solution was stored at −35 °C, affording large, dark purple crystals. Yield: 0.200 g, 55%. Screening by single-crystal XRD afforded lattice parameters matching those previously reported.[2]

1.3 Preparation of Magnetically Dilute 2

1 mg of **2** and 99 mg of $\mathbf{2_{Yb}}$ were dissolved in 4 mL THF then layered with 10 mL Et_2O and stored at −78 °C to afford deep green crystals. These crystals were darker than those of pure $\mathbf{2_{Yb}}$, consistent with incorporation of **2**.

Table S1: Collected crystal field parameters from fits of magnetometry for **1** and **2** in both the Wybourne and Stevens normalization. Only values that were varied in the fit are shown. The Stevens parameters are the raw outputs of fitting using the PHI program, from which the Wybourne parameters were converted. Stevens parameters from the pure-LS Hamiltonian for **2** cannot be compared to other parameters, and we did not convert them to the Wybourne normalization as the necessary $S = 3/2$, $L = 5$ prefactors have not been tabulated.

	**1** – Cp'_3Pr		**2** – Cp'_3Pr^-, Exchange		**2** – Cp'_3Pr^-, Pure-LS
	Wyb.	Stev.	Wyb.	Stev.	Stev.
B_2^0 (cm^{-1})	−3129(19)	23.18(15)	−950(60)	7(4)	19.6(4)
B_4^0 (cm^{-1})	1980(20)	−0.09533(11)	560(40)	−0.026(2)	−0.166(16)
B_6^0 (cm^{-1})	484(11)	0.000747(18)	484	0.000747	−0.00038(8)
B_6^6 (cm^{-1})	−1760(60)	−0.0412(14)	−1760	−0.0412	0.177(2)
TIP (emu mol^{-1})	–		0.001048(6)		0.00130(1)

2 Modeling of SQUID Magnetometry Data

Magnetometry data for **1** and **2** were taken from Ref.[3] by digitizing the χT plots from the paper's supporting information.

2.1 Crystal-Field Fitting of Magnetometry Data for 1

The magnetometry data for **1** was fit with the effective Hamiltonian in Equation S1:

$$\hat{\mathcal{H}}_{\mathrm{Pr^{III}}} = \mu_{\mathrm{B}}\vec{B} \cdot (k\hat{L} + 2\hat{S}) + k\lambda\hat{S} \cdot \hat{L} + \hat{V}_{C_{3h}}, \tag{S1}$$

where μ_B is the Bohr magneton, $\vec{B}$ is the externally applied magnetic field, $\hat{L}$ is the orbital angular momentum operator, $\hat{S}$ is spin angular momentum operator, λ is the spin-orbit coupling (SOC) constant, k is an orbital reduction factor, and $\hat{V}_{C_{3h}}$ is the crystal field (CF) operator with the idealized C_{3h} symmetry of a Cp_3Ln center. The spin system was defined for the ground LS multiplet (i.e., ground multiplet in the Russel-Saunders coupling scheme) of Pr^{III}, with $S = 1$, $L = 5$, and $\lambda = 380$ cm^{-1} (this latter value chosen as 90% of the free

atom value). The CF term of the Hamiltonian has 4 components, given by Equation S2:

$$\hat{V}_{C_{3h}} = \sum_{k=2,4,6} \sum_{\substack{q=0,6 \\ q \leq k}} B_k^q \hat{O}_k^q, \tag{S2}$$

where B_k^q are parameters and $\hat{O}_k^q$ are Wybourne operators. Note that while the PHI program[4] is parametrized using the Stevens formalism, the resulting parameters are not transferable between configurations. The conversion between these parameters is straightforward, and given by Equation S3:

$$B_k^q = \frac{A_k^q}{\theta_k \alpha_k^q}, \tag{S3}$$

where A_q^k is a Stevens CF parameter, θ_k is a term-specific operator equivalent factor in the $|L, m_L, S, m_S\rangle$ basis (provided in the PHI manual in Table 2.2.4), and α^k are ratios between Wybourne and Stevens parameters tabulated in Ref.[5] Fitting proceeded starting from the parameters in Ref.[6] and optimizing the orbital reduction parameter, which is not included in this model but here serves as a composite factor treating metal-ligand covalency, Slater repulsions, and configuration interaction (the latter two of these are included in the previous treatment explicitly, but are not otherwise accounted for in the present model). Subsequently, the B_k^q parameters with increasing k were iteratively optimized, i.e. in the first iteration B_2^0 was allowed to vary with other B_k^q fixed, then B_2^0 and B_4^0 were varied, and finally all four parameters were allowed to freely vary. The results, in both the Wybourne and Stevens formalisms, are summarized in Table S1.

2.2 Crystal-Field Fitting of Magnetometry Data for 2

CF fitting of the magnetometry data for **2** was performed using two effective Hamiltonians, termed here "exchange" and "pure-LS". Additionally, for both models, an empirical temperature-independent paramagnetism (TIP) component was needed to properly describe the data. This could reflect either a deficiency in our model, or problems with the experi-

mental data reduction (e.g. failure to account for a ferromagnetic impurity). In the former case, the most likely deficiencies in our model are TIP afforded by low-lying 5d CF excited states (i.e. states where the d-electron occupies an orbital other than $5d_{z^2}$) or other low lying configurations such as $4f^3$. The results of both treatments, in both the Wybourne and Stevens formalisms, are summarized in Table S1 and depicted graphically in Figure S1.

The exchange Hamiltonian is given by Equation S4:

$$\hat{\mathcal{H}}_{\mathrm{Pr^{II}}} = \mu_{\mathrm{B}}\vec{B} \cdot (k\hat{L} + 2\hat{S}_{\mathrm{4f}} + 2\hat{S}_{\mathrm{5d}}) + k\lambda\hat{S}_{\mathrm{4f}} \cdot \hat{L}_{\mathrm{4f}} - 2j\hat{S}_{\mathrm{4f}} \cdot \hat{S}_{\mathrm{5d}} + \hat{V}_{\mathrm{4f}} \tag{S4}$$

which differs from Equation S1 by the addition of an exchange term, $-2j\hat{S}_{\mathrm{4f}} \cdot \hat{S}_{\mathrm{5d}}$ with j an isotropic coupling constant. For this model two "sites" were defined, the first with $S = 1$, $L = 5$, and $\lambda = 380$ cm^{-1} (identical with **1**) and the second a simple $S = 1/2$ that is exchange coupled to the first site. The orbital reduction parameter was identical to the fit for **1**. Fitting proceeded by allowing B_2^0 and the empirical TIP term to vary with other B_k^q fixed; then TIP, B_2^0, and B_4^0 were varied with the 6th rank terms fixed.

The pure-LS Hamiltonian is identical to Equation S1, but the $\mathrm{Pr^{II}}$ model differs in the definition of the spin system, namely by use of $S = 3/2$ and $L = 5$, with the SOC unchanged at $\lambda = 380$ cm^{-1}. It is important to reiterate that the CF parameters from this model are not to be compared with those from any others reported here, as they operate on the full LS term as if all the electrons reside in the f-shell and not individually upon the 4f and 5d components. Regardless, we have chosen to report them here to illustrate the utility of a fully LS-coupled description of **2**, in particular for mapping between the $|L_{4f}, m_{L_{4f}}, S_{4f}, m_{S_{4f}}, S_{5d}\rangle$ basis output by the exchange model above and a $|J, m_J\rangle$ basis. Since the CF parameters for this model cannot be converted from other results, the initial parameters were chosen in a more *ad hoc* fashion. B_2^0 was set to 20 cm^{-1} while the other components were set to 0 cm^{-1}, in Stevens formalism. All crystal field parameters we subsequently optimized together with TIP. The first several states resulting from this simulation were ordered identically to those

from the exchange Hamiltonian, with similar state splitting.

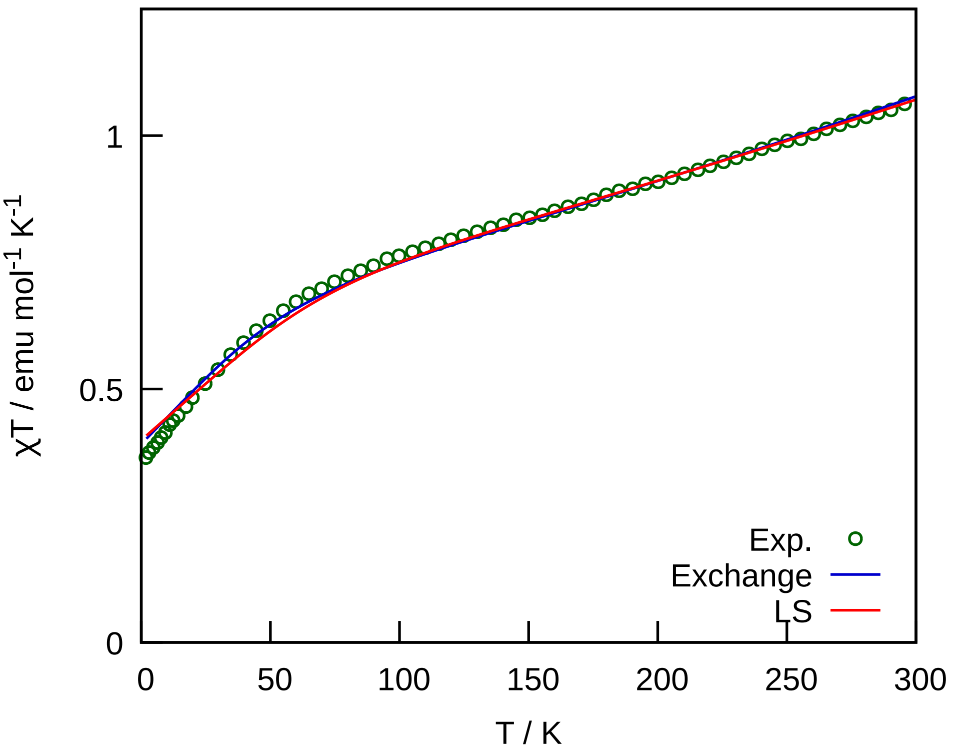

Figure S1: Comparison of simulations performed with the LS and Exchange Hamiltonians to experimental data for **2**. Magnetometry data were taken from Ref.[3] and are for a powder of undiluted **2**.

2.3 Crystal Field Approximation for 5d$_{z^2}$ Acting on a 4f Shell

For the exchange Hamiltonian treatment we chose to simplify the effect of the 5d$_{z^2}$/6s hybrid electron on the 4f shell by subsuming it in the ligand field term. In this treatment, the effect of the electron in the hybrid orbital is simply added to the potential generated by the ligands in the CF:

$$\hat{V}_{\text{total}} = \hat{V}_{\text{CF}} + \hat{V}_{\text{hyb}} = \sum_{k} \sum_{|q|\leq k} (B^q_{k,CF} + B^q_{k,hyb})\hat{O}^q_k, \tag{S5}$$

where $\hat{V}_{\text{hyb}}$ is the potential due to the electron in the hybrid orbital. To effectively use this approximation, it is necessary to determine which CF parameters should be effected by this added electron.

In general, a hybrid orbital can be expressed as an expansion in spherical harmonics as

given by Equation S6:

$$\psi_{\mathrm{hyb}}(r,\theta,\phi)=\sum_n\sum_l\sum_m c_l R_{n,l}(r)Y_l^m(\theta,\phi), \tag{S6}$$

where c_l are orbital coefficients, $R_{n,l}$ are radial components, and $Y_m^l(\theta,\phi)$ angular components of atomic orbitals with quantum numbers n,l,m. Since the orbital of interest is primarily an s/d_{z^2}-hybrid, it can be written as:

$$\psi_{\mathrm{sd}}(r,\theta,\phi)=c_0R_{6,0}(r)Y_0^0(\theta,\phi)+c_2R_{5,2}(r)Y_2^0(\theta,\phi), \tag{S7}$$

which is simply an explicitly-written spherical harmonic expansion truncated at $l=2$, omitting the term with $l=1$ and $c_l=0$.

Since V_{hyb} is generated by the charge distribution of the electron of interest, it should be determined by the probability density, $|\psi_{\mathrm{sd}}|^2$. This is simply the product of two spherical harmonic expansions. Such a product has a maximum rank of $k\leq k_1+k_2$, in this case 4. Thus, from Equation S8:

$$V_{\mathrm{hyb}}\propto|\psi_{\mathrm{sd}}|^2=\sum_{l=0}^{4}c_l'R_{n,l}(r)Y_l^0(\theta,\phi), \tag{S8}$$

here the new c_l' are given by Equation S9:

$$c_l'=\sum_{l_1=0}^{2}\sum_{l_2=0}^{2}c_{l_1}c_{l_2}Q_{l_1l_2}^{l}, \tag{S9}$$

with $Q_{l_1l_2}^{l}$ a Clebsch-Gordan coefficient (we have dropped the m-dependence here as they are all 0). Recognizing the similarity between Equation S8 and the CF Hamiltonian defined in terms of spatial coordinates, the radial component of Equation S8 can be grouped with c_l'

and any other proportionality and normalization factors necessary to define Equation S10:

$$\hat{V}_{\mathrm{hyb}} = \sum_{k=0}^{4} B_k^0 \hat{O}_k^0, \tag{S10}$$

where B_k^0 is a fitting factor and we have passed from the spherical harmonics Y_l^m through to the operator equivalents $\hat{O}_k^q$.

It is apparent given Equation S7 and S9 that the terms of Equation S10 will only be nonzero for $k = 0, 2, 4$, of which only 2 and 4 are important for a single configuration. Thus, under this approximation, the addition of an s/d-hybrid electron is expected to contribute to the second- and fourth-rank axial ($q = 0$) components of the CF.

3 X-band EPR Spectroscopy

X-band electron paramagnetic resonance (EPR) spectroscopy was performed using a Bruker Elexys E580 spectrometer equipped with a Bruker EN 4118X-MD4 X-band pulse-ENDOR resonator in an Oxford CF935 flow cryostat. Temperature was maintained with liquid helium cryogen and controlled by an Oxford ITC503 temperature controller. Samples were ground to a fine powder, loaded into 3 mm outer diameter quartz tubes that had been pre-sealed at one end, and then flame-sealed under vacuum. Spectra were recorded on 2 independently-synthesized samples of ca. 1% **2** diluted in $\mathbf{2_{Yb}}$ with similar results ($\mathbf{2_{Dil}}$). Data processing was performed either in MATLAB using the EasySpin (version 6.0.0-dev.51) toolbox[7] or the Bruker XEPR software provided with the spectrometer.

3.1 EDFS spectra and variable-field two-pulse ESEEM

Echo-Detected Field-Swept (EDFS) spectra were recorded using a primary echo sequence, i.e. $\pi/2 - \tau - \pi - \tau -$ echo. The $\pi/2$ pulse length was 16 ns and for π pulse 32 ns. For $\mathbf{2_{Dil}}$, it was found that severe Electron Spin Echo Envelope Modulation (ESEEM) modulation was present at low field (below ca. 300 mT) due to hyperfine coupling to ^{1}H nuclei on the ligands (*vide infra*). Through this field range, the rapid increase in the Larmor frequency of the ^{1}H nuclei gave rise to undesired structure in the EDFS spectra at constant delay times. To suppress this effect, spectra were determined from a variable-field variable-delay experiment, where τ was varied between 0.3 and 8.4 μs (512 points with a spacing of 16 ns). Above ca. 800 mT there were no observed spectral features, and the echo intensity at these fields was used to determine a constant baseline. The spectra were then normalized by setting the maximum recorded echo intensity to 1. The resulting ESEEM vs. B_0 spectra are shown in Figure S2 for each sample using different microwave frequencies. The resulting 2D spectrum was integrated in the time dimension to afford the final spectra with suppression of ESEEM artifacts as presented in the manuscript.

ESEEM processing was performed by fitting an exponential decay or stretched exponential decay to each field. After subtraction of the fit, the data were windowed with a Hamming function, zero filled to twice the total data length, and Fourier transformed (Figure S3). A strong feature tracking with the ^{1}H Larmor frequency was observed in all cases.

3.2 Relaxation time determinations

T_2 relaxation times were determined directly from the variable-delay EDFS measurement by least-squares fitting an exponential decay given by Equation S11 to the time axis data at a given field.

$$M_{xy} = M_0 e^{-t/T_2} \tag{S11}$$

T_1 relaxation time measurements were determined from an inversion recovery echo pulse sequence, i.e. $\pi - \tau - \pi/2 - t - \pi - t -$ echo, fit using an exponential recovery given by Equation S12:

$$M_z = M_0(1 - 2e^{-t/T_1}) + c \tag{S12}$$

where c is an offset that was necessary to properly fit the data. These results for 2 fields and 2 temperatures at 9.834 GHz microwave frequency are shown in Figure S4.

4 HF-EPR Spectroscopy

High-frequency electron paramagnetic resonance (HF-EPR) spectra were collected using a home-built transmission spectrometer described in detail previously,[8] operating in continuous wave (cw) mode with magnetic field modulation producing a derivative spectrum. The sample was measured as a ground polycrystalline undiluted powder immobilized in the EPR sample holder.

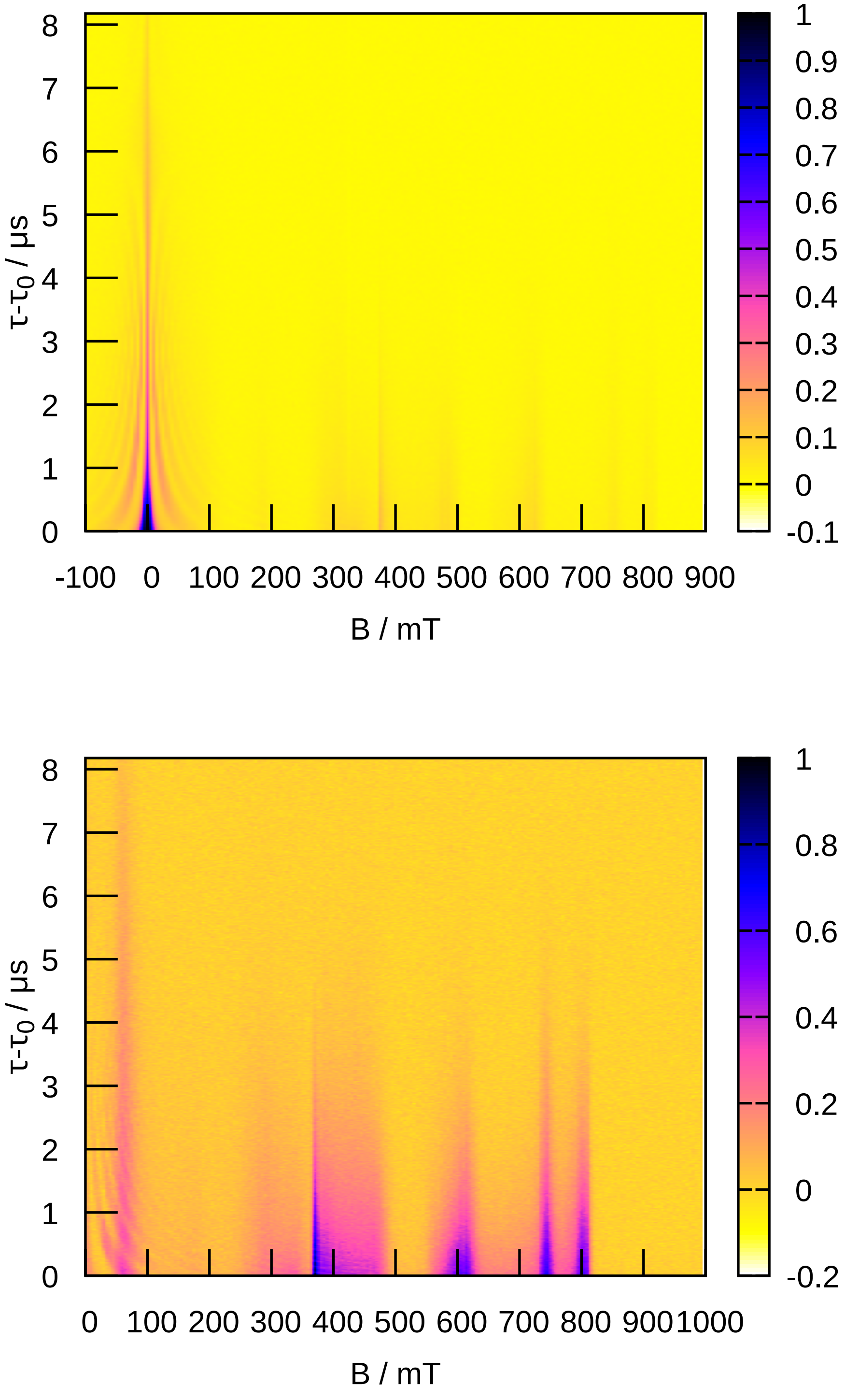

Figure S2: Normalized echo intensity vs. field for a sample of $\mathbf{2_{Dil}}$ at various delay times using a 2P-ESEEM sequence. Top: 9.834 GHz microwave frequency. Bottom: 9.723 GHz microwave frequency. These spectra represent independent sample preparations.

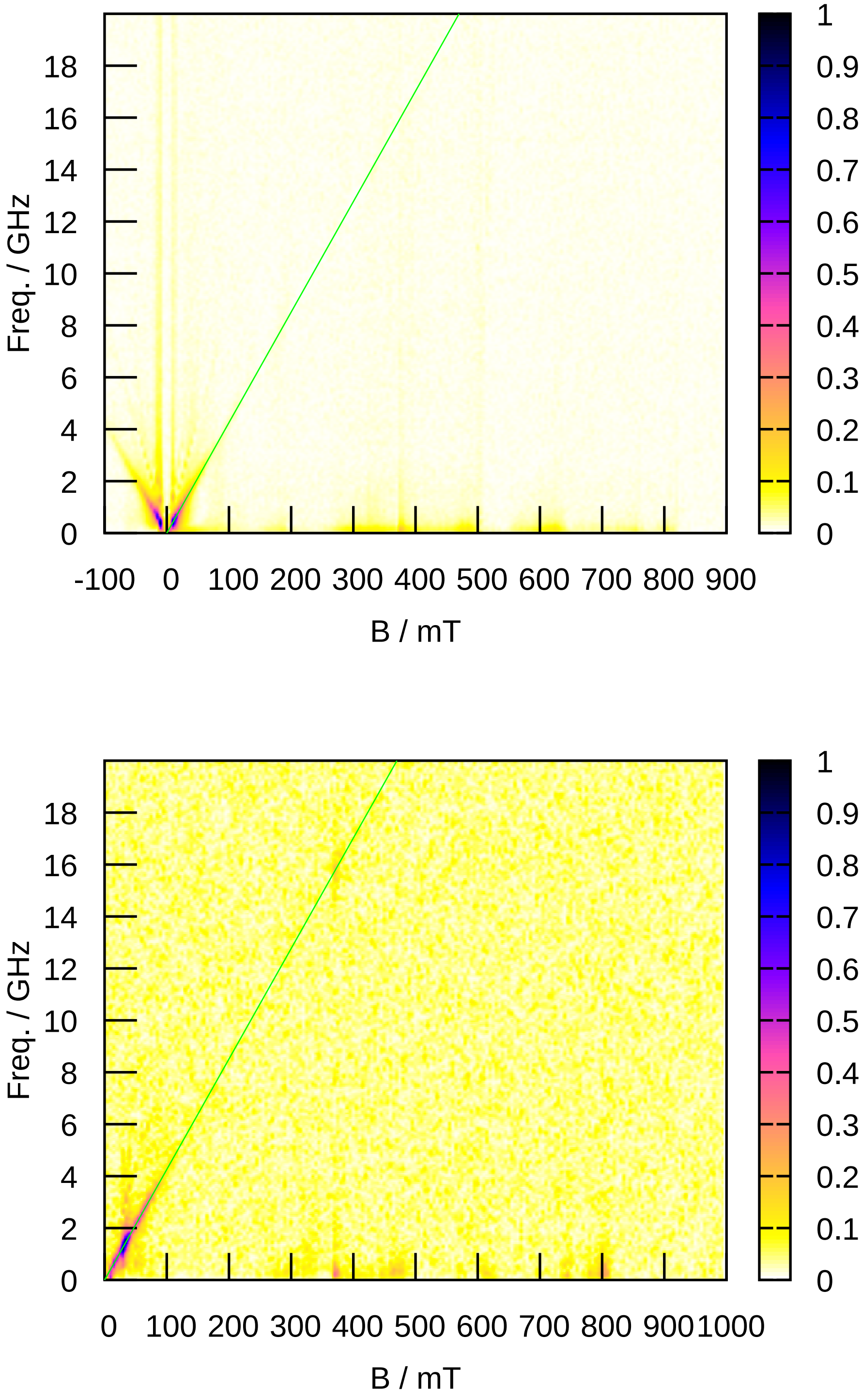

Figure S3: Normalized Fourier transform of processed data from Figure S2. A green line is drawn to represent the proton Larmor frequency. Top: 9.834 GHz microwave frequency. Bottom: 9.723 GHz microwave frequency. These spectra represent independent sample preparations.

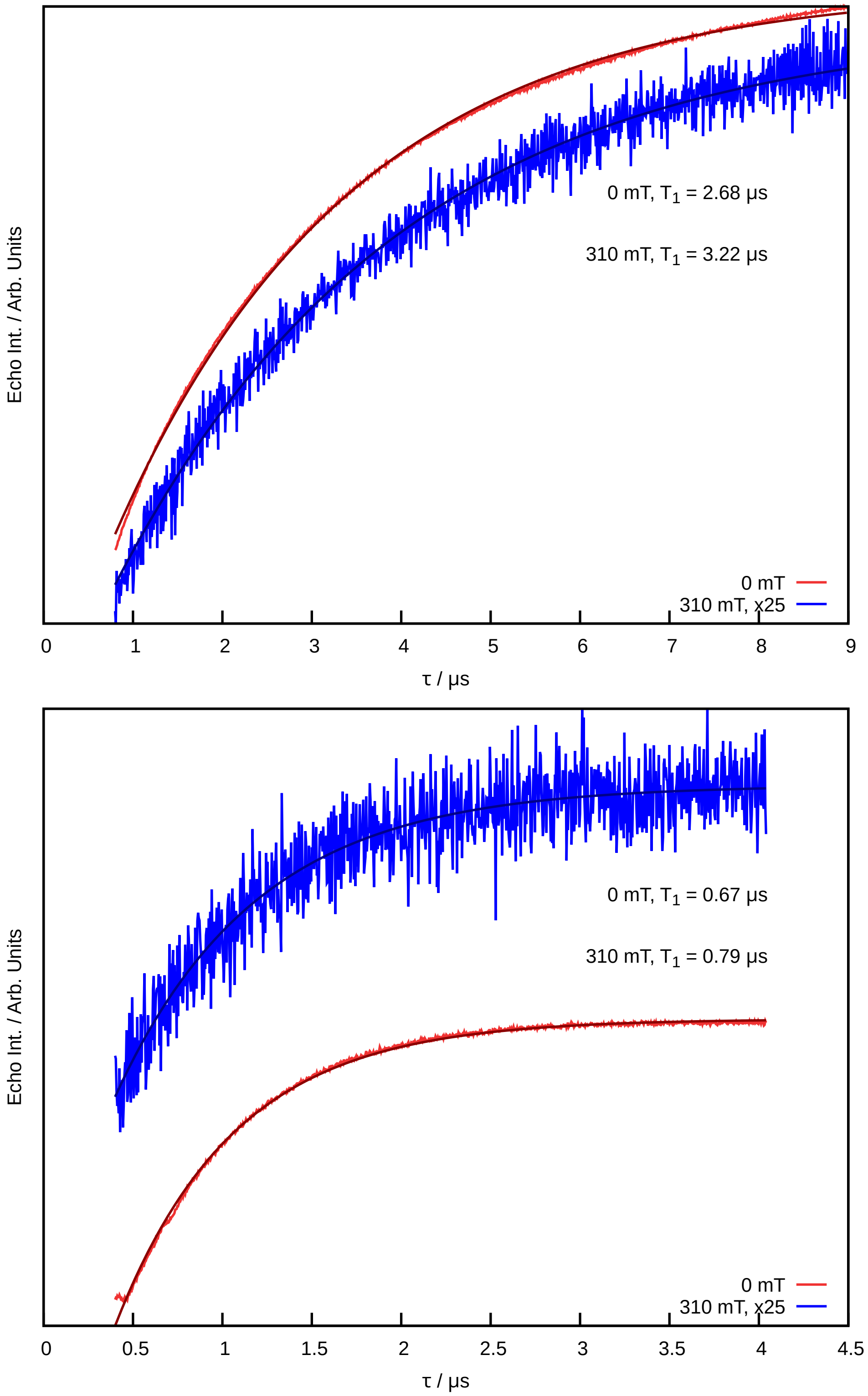

Figure S4: Inversion recovery curves for $\mathbf{2_{Dil}}$ at 5 K (top) and 10 K (bottom) at 0 and 310 mT. Echoes were not observed above 10 K. 310 mT data were rescaled for clarity.

5 EPR Simulation

5.1 Simulation of 70 GHz Continuous-Wave Spectra

The 70 GHz spectrum was simulated using exact diagonalization of Equation S13 using the EasySpin package (version 6.0.0-dev.51), a toolbox for Matlab,[7] with the following spin Hamiltonian (SH):

$$\hat{\mathcal{H}}_{\mathrm{EPR}} = \mu_{\mathrm{B}}\vec{B} \cdot \overleftrightarrow{g_{\mathrm{e}}} \cdot \hat{S} - \mu_{\mathrm{N}} g_{\mathrm{N}} \vec{B} \cdot \hat{I} + \hat{S} \cdot \overleftrightarrow{A} \cdot \hat{I}, \tag{S13}$$

where the first two terms respectively denote the electron and nuclear Zeeman interactions ($\overleftrightarrow{g_{\mathrm{e}}}$ is the electron g-tensor) and the third term represents the electron–nuclear hyperfine interaction ($\overleftrightarrow{A}$ is the hyperfine coupling tensor).

The importance of the 70 GHz selection needs to be pointed out as the hyperfine splitting is not resolved at higher frequencies (Figure S5) presumably due to g-strain. Immobilization of the sample also plays a crucial role in this study, as $g_{\|}$ features vanish for loose samples, presumably due to sample alignment with the magnetic field. Therefore, the optimal HF-EPR spectra were obtained at 70 GHz for immobilized powder samples.

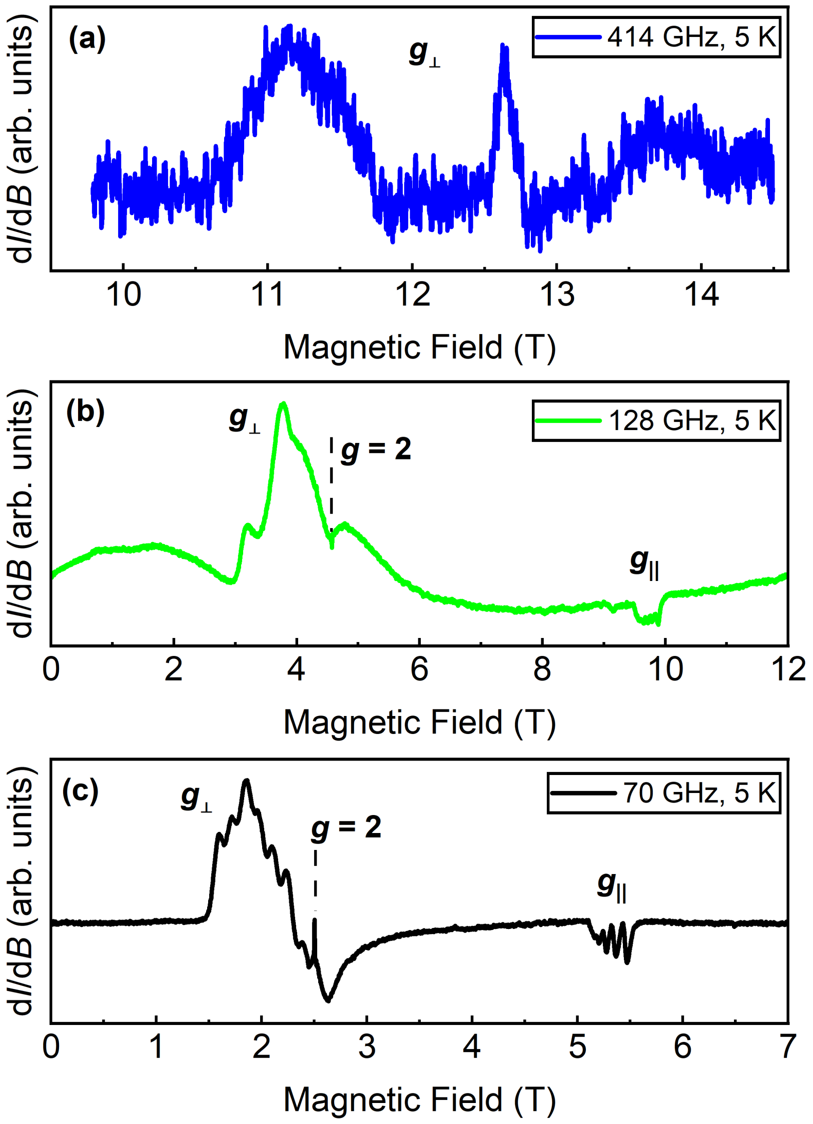

Figure S5: Frequency dependence of the 5 K cw HF-EPR spectra for **2** at: (a) 414 GHz, (b) 128 GHz, and (c) 70 GHz (see legend). The dashed line denotes the $g = 2$ position. At 414 GHz, the $g_{||}$ and $g = 2$ positions are beyond the maximum magnetic field of 14.8 T.

The temperature dependence of the cw-70 GHz spectra for **2** is shown in Figure S6. Remarkably, spectra are observed up to at least ambient temperature.

Table S2: Results of fitting the 70 GHz spectrum. All values other than g and g-strain (dimensionless) and line width (mT) reported are in MHz.

Param.	Value
A_x	4700
A_y	3700
A_z	870
g_x	2.60
g_y	2.21
g_z	0.94
A_x Strain	1000
A_y Strain	1000
A_z Strain	50
g_x Strain	0.05
g_y Strain	0.22
g_z Strain	0.00
Line width (peak-to-peak)	40 + 15 (Gaussian + Lorentzian) mT

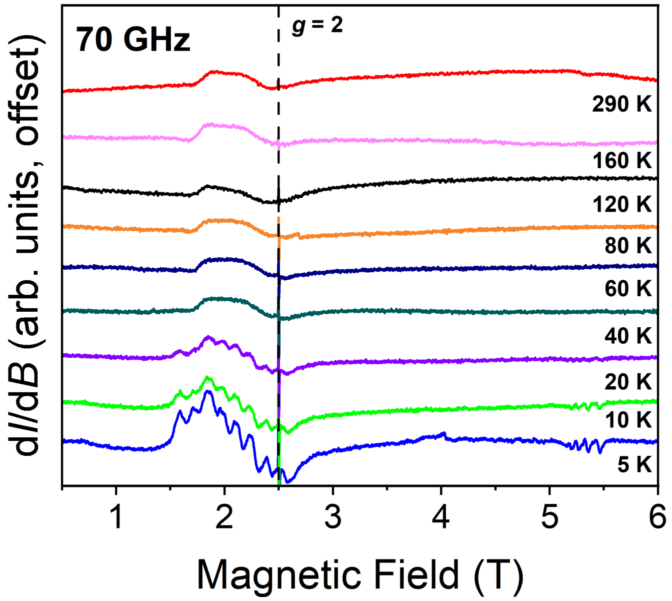

Figure S6: Temperature dependent cw HF-EPR spectra for **2**, recorded at 70 GHz and 5, 10, 20, 40, 60, 80, 120, 160, 290 K (see legend). The dashed line at 2.5 T denotes the $g = 2$ position.

The full set of 70 GHz simulation parameters (see Figure 3 in the main text) are collected in Table S2.

Table S3: Results of fitting to the X-band spectrum. All values other than g and g strain (which are dimensionless) and line width (in mT) reported are in MHz.

Param.	Value
$A_\perp$	4328
$A_\parallel$	872
$g_\perp$	2.43
$g_\parallel$	0.94
$A_\perp$ Strain	148
$A_\parallel$ Strain	51
$g_\perp$ Strain	0.07
$g_\parallel$ Strain	0.01
Isotropic broadening	8.9 mT

5.2 Simulation of X-band Spectra

X-band spectral simulation began with the parameters determined from the SH at 70 GHz as a starting point. For this simulation, the model was modified to use an axial SH rather than a rhombic one by taking the average of the x- and y-components of the g and A tensors to represent the $\perp$ components for an axial SH. Using these parameters, the initial simulated X-band spectrum, without the inclusion of any strains, is already in good agreement with the experimental spectrum, highlighting that the spectra taken at both frequencies likely reflect similar environments despite dramatically different concentrations of the magnetic site. In particular, at higher fields (above ca. 300 mT) the line positions and number of lines are similar between the experiment and this simulation. To improve the model, a least-squares fit using the ESFIT utility provided by EasySpin was performed, adding g- and A-strains to represent orientation-dependent broadening. The full X-band fit parameters are collected in Table S3.

References

(1) Fieser, M. E.; Macdonald, M. R.; Krull, B. T.; Bates, J. E.; Ziller, J. W.; Furche, F.; Evans, W. J. Structural, spectroscopic, and theoretical comparison of traditional vs recently discovered Ln^{2+} ions in the [K(2.2.2−cryptand)][($C_5H_4SiMe_3$)$_3$Ln] complexes: The variable nature of Dy^{2+} and Nd^{2+}. *Journal of the American Chemical Society* **2015**, *137*, 369–382.

(2) MacDonald, M. R.; Bates, J. E.; Ziller, J. W.; Furche, F.; Evans, W. J. Completing the Series of +2 Ions for the Lanthanide Elements: Synthesis of Molecular Complexes of Pr^{2+}, Gd^{2+}, Tb^{2+}, and Lu^{2+}. *J. Am. Chem. Soc.* **2013**, *135*, 9857–9868.

(3) Meihaus, K. R.; Fieser, M. E.; Corbey, J. F.; Evans, W. J.; Long, J. R. Record High Single-Ion Magnetic Moments Through $4f^n5d^1$ Electron Configurations in the Divalent Lanthanide Complexes $C_5H_4SiMe_3)_3Ln]^-$. *Journal of the American Chemical Society* **2015**, *137*, 9855–9860.

(4) Chilton, N. F.; Anderson, R. P.; Turner, L. D.; Soncini, A.; Murray, K. S. PHI: A powerful new program for the analysis of anisotropic monomeric and exchange-coupled polynuclear d- and f-block complexes. *Journal of Computational Chemistry* **2013**, *34*, 1164–1175.

(5) Newman, D. J.; Ng, B. In *Crystal Field Handbook*; Newman, D. J., Ng, B., Eds.; Cambridge University Press: Cambridge, UK, 2007; Chapter 2, pp 26–42.

(6) Jank, S.; Reddmann, H. S. Jank, H. Reddmann, H.-D. Amberger. *J. Alloys Compd.* **1997**, *250*, 387–390.

(7) Stoll, S.; Schweiger, A. EasySpin, a comprehensive software package for spectral simulation and analysis in EPR. *Journal of Magnetic Resonance* **2006**, *178*, 42–55.

(8) Hassan, A. K.; Pardi, L. A.; Krzystek, J.; Sienkiewicz, A.; Goy, P.; Rohrer, M.; Brunel, L. C. Ultrawide Band Multifrequency High-Field EMR Technique: A Methodology for Increasing Spectroscopic Information. *Journal of Magnetic Resonance* **2000**, *142*, 300–312.
